# From complaints to ethical-professional education: a study based on nursing ethical disciplinary proceedings

**DOI:** 10.1590/0034-7167-2024-0452

**Published:** 2025-12-08

**Authors:** Rafaela Serpa, Dulcinéia Ghizoni Schneider, Denise Elvira Pires de Pires, Silvana Alves Benedet, Felipa Rafaela Amadigi

**Affiliations:** I Universidade Federal de Santa Catarina, Hospital Universitário Polydoro Ernani de São Thiago. Florianópolis, Santa Catarina, Brazil; II Universidade Federal de Santa Catarina. Florianópolis, Santa Catarina, Brazil

**Keywords:** Ethics, Professional, Ethics, Nursing, Education, Nursing, Education, Continuing, Liability, Legal., Ética Profesional, Ética en Enfermería, Educación en Enfermería, Educación Continua, Responsabilidad Legal.

## Abstract

**Objectives:**

to identify elements that can foster ethical-professional education in nursing, based on the grounds for complaints in ethical disciplinary proceedings in Santa Catarina (2017-2021).

**Methods:**

a qualitative document-based study was carried out with data from 178 nursing ethical disciplinary proceedings, considering 33 grounds for complaints grouped into nine subcategories: Iatrogenic harm, Professional disagreements, Crimes, Assaults, Unauthorized practice, Use of social media, Inadequate medical record documentation, Unprofessional behavior, and Abandonment of duty. Content analysis was applied to systematize the data.

**Results:**

the articulation between the grounds for complaints and the socioemotional competencies of Education 5.0 revealed that strengthening these competencies may prevent unethical conduct and help identify elements to improve ethical-professional education.

**Final Considerations:**

the socioemotional competencies of Education 5.0 equip nurses with essential skills, suggesting an innovative path to reduce errors and promote a more ethical and responsible professional practice.

## RESUMEN

**Objetivos:**

identificar elementos que impulsen la educación ético-profesional en enfermería a partir de los motivos de denuncia de procesos éticos concluidos en Santa Catarina (2017-2021).

**Métodos:**

estudio cualitativo documental con datos de 178 procesos éticos de enfermería. Se consideraron 33 motivos de denuncia agrupados en nueve subcategorías: Iatrogenias, Desavenencias profesionales, Delitos, Agresiones, Ejercicio ilegal de la profesión, Uso de redes sociales, Registros inadecuados en la historia clínica, Actitudes profesionales inadecuadas y Abandono de turno. Para sistematizar los datos, se aplicó análisis de contenido.

**Resultados:**

la articulación de los motivos de denuncia con las competencias socioemocionales de la Educación 5.0 mostró que fortalecer dichas competencias puede prevenir conductas no éticas, identificando elementos para perfeccionar la educación ético-profesional.

**Consideraciones Finales:**

las competencias socioemocionales de la Educación 5.0 proporcionan habilidades esenciales para profesionales de enfermería, sugiriendo un camino innovador para reducir errores y promover una práctica profesional más ética, segura y responsable.

## INTRODUCTION

Nursing is a profession committed to human care in all its dimensions. This care integrates technical-scientific and theoretical-philosophical knowledge with the ethical-legal precepts of the profession, which are essential for the functioning of healthcare services in the practices of providing care, educating, managing, teaching, and conducting research^(1)^. Such responsibility requires nurses to generate knowledge that supports daily actions and to prepare new professionals with the competence and knowledge to face the challenges of a constantly changing world^(2)^.

This change, driven by technological advances-such as artificial intelligence and robotics, which perform tasks traditionally carried out by humans-introduces a new concept: Society 5.0. In this scenario, where people will work in activities that do not yet exist, the educational system must evolve, adopting an innovative and technological approach^(3)^.

However, the educational process is complex. Although moral and ethical development is not restricted to educational institutions, its relevance in this process is undeniable. Since ethical commitment must be ongoing and begin during professional training, the role of educators is essential in this trajectory, preparing professionals to work in different areas of nursing with technical, ethical, and political competence^(4)^.

Continuing health education, a sense of belonging to the nursing profession, and teamwork are seen as strategies to strengthen professional recognition and visibility^(5)^. Other authors emphasize that the continuing education of nursing teams is a key factor in preventing and reducing errors, as well as in improving healthcare services^(6)^.

In this regard, Silva^(7)^ highlights that various underlying causes contribute to unethical events in nursing practice, making it necessary for technical and scientific training to be accompanied by ethical knowledge in order to reinforce the responsibilities, rights, and duties of the profession.

When viewed in this context, it is crucial to develop strategies for the ethical-professional education of nursing students. Ethical education is dynamic and ongoing, and schooling and professional training play an important role in this process. When students become professionals, they develop a collective sense of belonging and acquire guiding references^(8)^.

Within this process, Education 5.0 indicates that universities and schools, in addition to revolutionizing and preparing citizens for the world of work, must also prepare them to live in society, to act ethically and responsibly, and to use technological tools to build a more inclusive, productive, and ethical society that guarantees rights and respects humanity^(9)^.

This new educational model is the outcome of at least 180,000 years of technological progress: from cave paintings to the mastery of fire and agriculture, trade routes and voyages, the invention of the printing press, the emergence of universities, telecommunications, the internet, and the human psyche, culminating in the most refined art developed by humankind-education^(10)^. Such an approach values comprehensive training, student participation, and the human essence^(9)^.

This teaching strategy fosters skills and competencies by combining knowledge, values, and attitudes. Soft skills, fundamental to human development, are central in this context and include ethics, empathy, communication skills, problem-solving, teamwork, emotional regulation, and valuing diversity^(9,11)^.

The ethical training of nurses must keep pace with changes in education, considering the technological and communication innovations that shape the contemporary world^(12)^. Therefore, education must adapt to these transformations to ensure professional practice is aligned with current demands.

## OBJECTIVES

To identify elements that can contribute to ethical-professional education, based on the analysis of the grounds for complaints in nursing ethical disciplinary proceedings at the Regional Nursing Council of Santa Catarina, concluded between 2017 and 2021.

## METHODS

### Ethical considerations

The study adhered to the guidelines of Resolution No. 466/2012 of the National Health Council and Resolution No. 510/2016. It was approved at the 610th Plenary Ordinary Meeting (ROP) of Coren-SC. Although ethical approval was not required because the study used publicly available data, it was submitted to the Research Ethics Committee of the Federal University of Santa Catarina (UFSC).

### Study design

This is a qualitative, document-based study, using data produced in the context of a master’s thesis in nursing, linked to a larger research project coordinated by the thesis advisor. The analysis presented in this article supports the proposal of elements that may contribute to strengthening ethical professional education in nursing.

### Study setting

The study was conducted at the Regional Nursing Council of Santa Catarina (Coren-SC).

### Data source

Data were obtained from the Transparency Portal on the Coren-SC website. The documents analyzed were the minutes containing information on nursing ethical disciplinary proceedings processed and concluded by the Council between 2017 and 2021.

### Data collection and organization

Between March and June 2022, data were collected from 178 nursing ethical disciplinary proceedings, which were organized into six categories. One of these was entitled “Grounds for complaints”, comprising 33 items grouped into nine subcategories. For this study, the category “Grounds for complaints” was selected to be associated with the socioemotional competencies proposed in Education 5.0.

### Data analysis

Bardin’s Content Analysis^(13)^ was applied to systematize the data collected from the procedural documents. The category “Grounds for complaints” comprised nine subcategories, described below. The presentation of the study was guided by the Consolidated Criteria for Reporting Qualitative Research (COREQ).

## RESULTS

A total of 178 nursing ethical disciplinary proceedings were processed and concluded over a five-year period. The analysis of these proceedings defined six categories: 1) Complainants, 2) Respondents, 3) Grounds for complaints, 4) Violated articles of the Code of Ethics, 5) Case outcomes, and 6) Processing time.

The category “Grounds for complaints” was organized into 33 items grouped into nine subcategories: 1) Iatrogenic harm (medication-related or not), not associated with negligence, malpractice, or recklessness, 2) Professional disagreements, 3) Various crimes, 4) Assaults and mistreatment, 5) Unauthorized practice, 6) Use of social media, 7) Medical record documentation, 8) Unprofessional behavior, and 9) Abandonment of duty.

Following the analysis of this category, several grounds for complaints were selected to illustrate how these unethical events occur in nursing practice (Chart 1).

**Chart 1:** Grounds for complaints - Ethical disciplinary proceedings, Coren-Santa Catarina, Brazil, 2017-2021

GROUNDS FOR COMPLAINTS
- Workplace bullying
- Insubordination
- Abuse of power
- Abandonment of duty
- Disrespect for hierarchy
- Conflict of competencies
- Defamation of colleagues
- Racial prejudice against a colleague
- Unauthorized absence from the unit
- Failure in interpersonal communication
- Sharing patient photos on social media
- Physical and verbal assault of a colleague
- Misappropriation of medications and supplies

**Figure 1:**
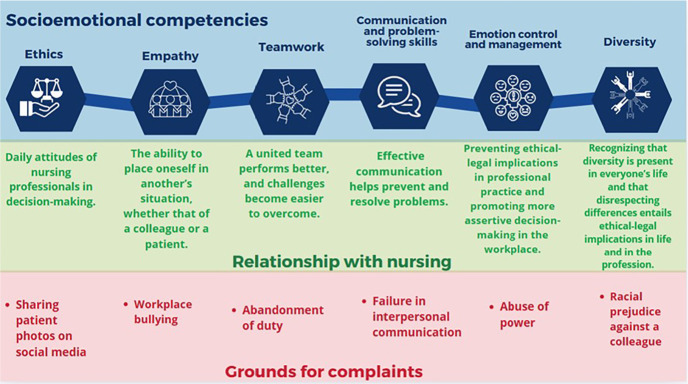
Grounds for complaints related to socioemotional competencies, based on Felcher et al^(11)^

The analysis of these unethical events led to the development of Figure 1, which shows the relationship between the grounds for complaints and socioemotional competencies. This analysis aimed to demonstrate that failures leading to complaints are more likely to occur when professionals do not consider or have not strengthened their socioemotional competencies.

The relationship between socioemotional competencies and nursing practice was established from the analysis of the main grounds for complaints that required professionals to respond to ethical disciplinary proceedings in their professional council. These situations can be used as learning opportunities, guiding professionals on the importance of values associated with socioemotional competencies in facing the daily challenges of the profession. When incorporated into practice, these attitudes significantly contribute to improving work quality, interpersonal relationships, and decision-making, thereby promoting more ethical, safe, and responsible nursing care.

**Figure 2:**
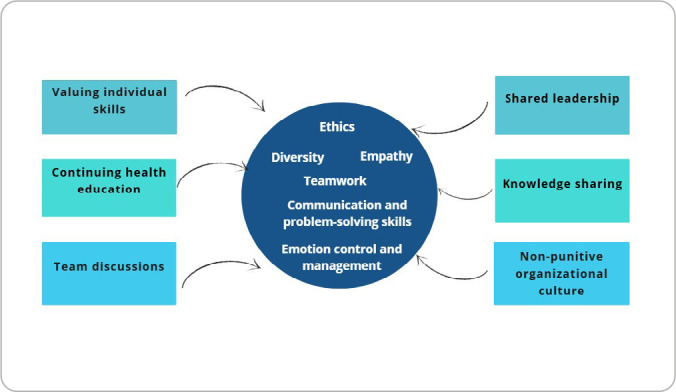
Proposed elements for developing socioemotional competencies

Socioemotional competencies enhance professional performance and interpersonal relationships in everyday practice. Considering this relationship with nursing, several elements were proposed to help professionals develop or strengthen such competencies (Figure 2).

## DISCUSSION

Errors in nursing practice arise from factors that require reflection, particularly the working conditions to which professionals are exposed: excessive workload, staff shortages, high turnover, and shortcomings in the organization and management of care. In light of these structural and organizational challenges, strategies must be considered to prevent errors that may harm patients and their families, including educational approaches that emphasize the ethical dimension of care^(14)^.

In nursing practice, beyond the technical-scientific knowledge acquired during training, it is essential to understand the laws, codes, and regulatory standards governing the profession. However, when examining the concept of Education 5.0, other equally relevant elements for ethical-professional education emerge, such as empathy, social responsibility, collaboration, and emotional intelligence. Although often absent from traditional curricula, these competencies directly influence the quality of care and the ethical stance of professionals.

When analyzing the relationship between the socioemotional competencies proposed by Education 5.0 and the grounds for complaints in ethical disciplinary proceedings, the impact of these skills on nursing practice becomes evident. Even though they cannot be quantified or formally recorded in curricula, such competencies make a decisive difference in both personal and professional life, as they combine competence, skill, and attitude^(9)^. This knowledge distinguishes the professional^(15)^.

Among the socioemotional competencies neglected by professionals in the analyzed proceedings, ethics stands out as a social instrument that guides human behavior toward living well in society^(16)^. All grounds for complaints revealed shortcomings in this competency; however, for illustrative purposes, each skill was associated with a theme that presented a clearer relationship.

Ethics as a competency arises from the individual opportunity to choose actions, which generates unique responsibility^(17)^. For example, when complaints concerning the sharing of patient photos on social media or other issues cited in the study are confirmed, professionals are violating ethical principles.

Therefore, every decision-making process requires reflection grounded in reason and ethical principles; otherwise, individuals are more likely to commit infractions. It is urgent to develop educational programs that address the types of errors and their causes, discussing problem scenarios and proposals for improvement. The nursing team, often held responsible for errors, fears judgment and retaliation, which leads to underreporting and failures in following up on situations that incorrectly result in errors^(18)^.

Another neglected competency was empathy. Encompassing both cognitive and emotional capacities, it is crucial in nursing, especially for understanding and connecting with the feelings of others^(19)^. Its absence may lead to harmful actions, such as abandonment of duty or the inappropriate sharing of patient photos on social media. Other authors emphasize empathy as vital to health, underscoring the need for reforms in the organizational culture of healthcare institutions to improve performance and well-being in the workplace^(20)^.

Communication and problem-solving skills also showed shortcomings. Deficiencies in this area can compromise patient safety and team harmony. In this study, the impairment of this competency was associated with the ground “Failure in interpersonal communication”. For example, shift handover is a decisive moment for communication between nursing teams, as effective information exchange ensures continuity and quality of care, both of which are essential for patient safety^(21)^.

Therefore, effective communication is not only fundamental to promoting patient safety but also to resolving conflicts and fostering flexibility and teamwork^(22)^. Professional experience and values, combined with the ability to develop strategies focused on safeguarding patients, form the core of decision-making in nursing care^(23)^.

Teamwork is another socioemotional competency that supports good performance within this new educational model. In this study, this skill was compromised when a professional abandoned duty. Some authors^(22,24)^ highlight interpersonal relationships and teamwork in nursing as fundamental factors; when aligned with effective management strategies, they not only address challenges and improve outcomes but also enhance staff motivation and performance. Teamwork underpins the provision of safe care, promoting cohesion as well as the sharing of knowledge and goals, which directly improves care quality and satisfaction among both staff and patients.

Problems in emotion control and management were also associated with the grounds for complaints, such as in cases of abuse of power. Effective emotion management is highlighted as a key competency in nursing, particularly in contexts of abuse of power. Emotional intelligence-understood as the ability to understand and manage one’s own feelings and those of others-plays a decisive role in conflict resolution, goal achievement, and the maintenance of healthy interpersonal relationships in the workplace^(25,26)^. In nursing leadership, applying this ability is indispensable to mitigating team emotional strain and promoting compassionate and effective care practices^(27)^.

It is necessary to invest in continuing education initiatives that prepare nurses to manage conflicts. Training plays a key role in developing competencies such as interpersonal relationships and communication, and this topic should also be addressed during undergraduate education^(28)^.

Diversity, recognized as an essential socioemotional competency, is particularly relevant in nursing in cases of complaints of racial prejudice. Reflecting the concept of a broad spectrum of human differences, it encompasses professional, generational, ability-related, sexual orientation, and religious belief aspects^(29)^. Driven by the pursuit of a more just society, diversity has been increasingly acknowledged in different contexts, leading to significant change^(30)^.

Discussing diversity means exploring the multifaceted aspects of human experience, encompassing personal, cultural, and historical characteristics. Such reflection helps us better understand ourselves and others, granting due respect to individual differences. Professionals who value diversity contribute to building a more inclusive and ethical environment in their practice^(30)^.

In the 21st century, educational programs must prepare professionals who are reflective, critical, and capable of driving change-not merely occupying a position, but acting as true agents of transformation^(11)^. Along with technological advancements, the future of society is intrinsically tied to the development of strong ethical practices^(31)^.

In this regard, Education 5.0 emerges as an innovative model for preparing individuals to enter the labor market, equipping them with the socioemotional competencies required to be collaborative professionals committed to the collective good, thereby fostering a more altruistic society^(32)^. Although some of these competencies are already familiar in both professional and personal spheres, they now take on a prominent role in this new context.

From this perspective, it is required to prepare professionals for ethical, creative, reflective, and problem-solving practice, considering that these competencies integrate knowledge, skills, and attitudes, shaping professional conduct. Knowledge alone is not sufficient; it must be translated into the skills and attitudes of professionals.

With this in mind, several elements were proposed to strengthen socioemotional competencies: valuing individual skills, team discussions, shared leadership, a non-punitive organizational culture, knowledge sharing, and continuing health education.

When individual skills are valued, professionals feel recognized within the team, which encourages knowledge sharing and teamwork. This element is associated with team discussions, which play a central role in healthcare services by fostering unity among professionals and broadening perspectives on problems, since each professional offers a different viewpoint, contributing to service improvement.

Regarding leadership, it is important for leaders to listen to their teams, share ideas, and take group opinions into account. By doing so, they are more likely to make assertive decisions and earn the respect and admiration of their teams, fostering a healthy and empathetic work environment. In a study conducted to assess patient safety in a philanthropic hospital in Minas Gerais, it was observed that effective communication among professionals and leaders’ ability to listen to their teams favored safer care^(33)^.

Another proposed element is the development of a non-punitive organizational culture in healthcare services. Such a culture creates an environment conducive to reporting adverse events, and knowledge of these errors makes it possible to implement improvements, such as developing protocols and changing service routines. In a study assessing patient safety culture from the perspective of nursing professionals, the importance of not treating errors punitively was highlighted, as a punitive climate leads to fear of reporting. Instead, the authors recommend that errors be analyzed from an educational perspective^(34)^.

Continuing health education is also proposed as an element to strengthen competencies. It is considered one of the main strategies for reducing errors in nursing. Education reinforces all the other elements, since knowledge is power: it empowers professionals to engage more effectively in team discussions, to exercise better leadership, to share and disseminate knowledge, and to value individual skills.

### Study limitations

The accessed documents did not provide detailed information about the specific locations where the events described in the ethical proceedings occurred-whether hospitals, primary healthcare units, clinics, or other services. Such information could have supported more in-depth analyses of institutional contexts and their possible influence on ethical conduct. Moreover, the documentary nature of the study limits the understanding of intentionality or the subjective circumstances involved in the analyzed episodes.

### Contributions to the field of nursing, healthcare, or public policy

The findings of this study strengthen ethical-professional education in nursing by identifying, from real ethical proceedings, formative elements aligned with the socioemotional competencies proposed by Education 5.0. These results provide support for improving academic training and implementing continuing health education strategies in healthcare services. In addition, they may inform institutional policies and management actions aimed at fostering more ethical, safe, and humanized professional practice.

## FINAL CONSIDERATIONS

The grounds for the ethical proceedings analyzed in this study highlight the need to reflect on which knowledge, skills, and attitudes remain fragile or require further development. Such reflections encourage the development of measures to reduce unethical behavior among nursing professionals. The findings also reinforce the importance of continuous and structured investment in ethical-professional education throughout training and professional practice.

It is undeniable that professional training, continuing health education, and knowledge of laws, codes, regulations, and resolutions influence professional practice, directly shaping how procedures are communicated, performed, and decided upon in both clinical and ethical dimensions. In this regard, the socioemotional competencies proposed by Education 5.0, discussed in this study, introduce essential skills into professional training, contributing to more ethical, collaborative, and responsive nursing practices that address contemporary demands.

Thus, strengthening competencies such as ethics, empathy, teamwork, emotion control and management, communication, problem-solving skills, and valuing diversity can foster safer, more reflective, and more responsible practices, grounded in the Ethics of Care. Professionals who cultivate these competencies tend to provide care with greater awareness of their social role, promoting healthy interpersonal relationships and actively contributing to the advancement of nursing and healthcare services.

## Data Availability

The research data are available only upon request.
