# Supplementary material for: From complaints to ethical-professional education: a study based on nursing ethical disciplinary proceedings
Source: Rev Bras Enferm. 2025 Dec 8;78(6):e20240452. doi: 10.1590/0034-7167-2024-0452 (PMC12707537; doi:10.1590/0034-7167-2024-0452)
Supplement: Translation

# Translation: Da denúncia à educação ética profissional: um estudo fundamentado em processos éticos de enfermagem

**DOI:** 10.1590/0034-7167-2024-0452pt

**Authors:** Rafaela Serpa, Dulcinéia Ghizoni Schneider, Denise Elvira Pires de Pires, Silvana Alves Benedet, Felipa Rafaela Amadigi

**Affiliations:** I Universidade Federal de Santa Catarina, Hospital Universitário Polydoro Ernani de São Thiago. Florianópolis, Santa Catarina, Brasil; II Universidade Federal de Santa Catarina. Florianópolis, Santa Catarina, Brasil

**Keywords:** Ética Profissional, Ética em Enfermagem, Educação em Enfermagem, Educação Continuada, Responsabilidade Legal.

## Abstract

**Objetivos:**

identificar elementos para impulsionar a educação ético-profissional em enfermagem, baseando-se nos motivos de denúncia dos processos éticos em Santa Catarina (2017-2021).

**Métodos:**

pesquisa qualitativa do tipo documental desenvolvida com dados de 178 processos éticos de enfermagem, considerando 33 temas de denúncias em nove subcategorias: Iatrogenias, Desavenças profissionais, Crimes, Agressões, Exercício ilegal da profissão, Uso de redes sociais, Registros inadequados em prontuário, Atitudes profissionais inadequadas e Abandono de plantão. Utilizou-se análise de conteúdo na sistematização dos dados.

**Resultados:**

a articulação dos temas de denúncias com as competências socioemocionais da Educação 5.0 revelou que fortalecer essas competências pode prevenir condutas não éticas, identificando elementos para aprimorar a educação ético profissional.

**Considerações Finais:**

as competências socioemocionais da Educação 5.0 oferecem habilidades essenciais para enfermeiros, sugerindo um caminho inovador para reduzir erros e promover uma prática profissional mais ética e responsável.

## INTRODUÇÃO

A enfermagem é uma profissão comprometida com o cuidado humano em todas as suas dimensões. Esse cuidado compreende conhecimentos técnico-científicos e teórico-filosóficos integrados com preceitos ético-legais da profissão, indispensáveis para o funcionamento dos serviços de saúde na prática de assistir, educar, gerenciar, ensinar e pesquisar^(1)^. Tal responsabilidade requer dos profissionais a produção de conhecimentos que fundamentem as ações diárias e formem novos profissionais com conhecimento e competência para enfrentar os desafios de um mundo em permanente transformação^(2)^.

Essa mudança decorrente dos avanços tecnológicos - como a inteligência artificial e a robotização, que executam tarefas usualmente realizadas pelo ser humano - traz um novo conceito: a Sociedade 5.0. Nesse cenário, em que as pessoas trabalharão em atividades ainda não existentes, o sistema educacional precisa evoluir, assumindo uma educação inovadora e tecnológica^(3)^.

No entanto, o processo de educar é uma atividade complexa. Embora a construção moral e ética não se restrinja às instituições de ensino, é inegável a sua relevância nesse processo. Considerando que o compromisso ético precisa ser permanente e iniciar durante a formação profissional, é imprescindível o papel do docente nesse percurso, educando esses profissionais para atuarem nas diferentes áreas da profissão, com competência técnica, ética e política^(4)^.

A educação permanente em saúde, o sentimento de pertencimento à profissão de enfermagem e o trabalho em equipe são observados como estratégias para o reconhecimento e visibilidade profissional^(5)^. Outros autores destacam que a formação contínua da equipe mostra-se como indicador relevante na prevenção e diminuição de erros, assim como referência de melhoria do serviço de saúde^(6)^.

Com esse mesmo olhar, Silva^(7)^ pontua que diversas são as causas subentendidas aos eventos não éticos no exercício profissional da enfermagem, sendo necessário que a formação técnica e científica venha acompanhada de um saber ético, para aprimorar as responsabilidades, direitos e deveres da profissão.

Nesse contexto, é preciso elaborar estratégias de educação ético-profissional dos estudantes de Enfermagem. É dinâmica e permanente a construção da educação ética, dentro da qual a escolarização e a formação do profissional exercem um papel importante. Quando o estudante passa a ser profissional, ele desenvolve um sentimento de pertencimento coletivo, adquirindo referências que o orientam^(8)^.

Nesse processo, a Educação 5.0 demonstra que as universidades e escolas, para além de revolucionar e formar cidadãos preparados para o mundo do trabalho, precisam prepará-los para saber conviver em sociedade, sendo éticos e responsáveis e utilizando as ferramentas tecnológicas para criar uma sociedade mais inclusiva, produtiva e ética, com garantia de direitos e respeito à humanidade^(9)^.

Esse novo modelo de ensino é uma conquista de pelo menos 180 mil anos de evolução da tecnologia: partiu-se das pinturas rupestres, passando pelo domínio do fogo e da agricultura, viagens e rotas comerciais, imprensa, início das universidades, telecomunicações, internet e psiquê humana, até alcançar a mais preciosa arte aprimorada pela humanidade - educação^(10)^. Uma nova educação como esta valoriza a formação integral, o aluno participativo e a essência humana^(9)^.

Tal estratégia de ensino trabalha as habilidades e competências por meio da associação de saberes, conhecimentos e atitudes. As *soft skills*, fundamentais para o desenvolvimento humano, são centrais nesse contexto, abrangendo ética, empatia, habilidades de comunicação, resolutividade de problemas, trabalho em equipe, gestão emocional e valorização da diversidade^(9,11)^.

A formação ética dos profissionais de enfermagem precisa acompanhar essas mudanças na educação, considerando as inovações tecnológicas e de comunicação que moldam o mundo contemporâneo^(12)^. Portanto, é essencial que o ensino se adapte a essas transformações para garantir uma prática profissional alinhada com as demandas atuais.

## OBJETIVOS

Identificar elementos que contribuam com a educação ético-profissional, com base na análise dos motivos/temas geradores dos processos éticos de enfermagem do Conselho Regional de Enfermagem de Santa Catarina concluídos entre 2017 e 2021.

## MÉTODOS

### Aspectos éticos

A pesquisa seguiu as orientações da Resolução nº 466/2012 do Conselho Nacional de Saúde, bem como a Resolução nº 510/2016. Foi realizada mediante a aprovação na 610ª Reunião Ordinária do Plenário (ROP) do Coren-SC. Embora dispensasse apreciação ética por utilizar dados de domínio público, a pesquisa foi submetida ao comitê de ética da Universidade Federal de Santa Catarina (UFSC).

### Tipo de estudo

Trata-se de uma pesquisa qualitativa de natureza documental, cujos dados foram produzidos no contexto de uma dissertação de mestrado em enfermagem, vinculada a um macroprojeto de pesquisa coordenado pela orientadora do trabalho. A análise realizada neste artigo fundamenta a proposição de elementos que podem contribuir para o fortalecimento da educação ético-profissional na enfermagem.

### Cenário do estudo

A pesquisa foi efetuada no Conselho Regional de Enfermagem de Santa Catarina (Coren-SC).

### Fonte de dados

A fonte de coleta de dados foi o Portal Transparência, no site do Coren-SC. Os documentos analisados foram as atas que contêm os dados dos processos ético-profissionais tramitados e concluídos no Conselho entre 2017 e 2021.

### Coleta e organização dos dados

De março a junho de 2022, foram coletados dados de 178 processos éticos, analisados sob a forma de seis categorias. Uma delas foi denominada “Motivos geradores das denúncias”, organizada em 33 temas, os quais foram agrupados em nove subcategorias. Para este estudo, foi selecionada a categoria “Motivos geradores das denúncias” para se fazer a associação com as competências socioemocionais propostas na Educação 5.0.

### Análise dos dados

A Análise de Conteúdo de Bardin^(13)^ foi utilizada para sistematizar os dados coletados nos documentos processuais. A categoria “Motivos geradores das denúncias” apresentava nove subcategorias, descritas a seguir. A estrutura de apresentação do estudo foi orientada pelos critérios do *Consolidated Criteria for Reporting Qualitative Research* (COREQ).

## RESULTADOS

Os 178 processos éticos foram tramitados e concluídos ao longo de cinco anos. O estudo dos processos definiu seis categorias: 1) Denunciantes, 2) Denunciados, 3) Motivo da denúncia, 4) Artigos infringidos do Código de Ética, 5) Desfecho do processo e 6) Tempo de tramitação.

A categoria “Motivos geradores das denúncias” foi organizada em 33 temas, agrupados em nove subcategorias: 1) Iatrogenias medicamentosas ou não associadas à negligência, imperícia ou imprudência, 2) Desavenças entre profissionais, 3) Crimes diversos, 4) Agressão e maus-tratos, 5) Exercício ilegal da profissão, 6) Redes sociais, 7) Registro em prontuário, 8) Atitudes profissionais inadequadas; e 9) Abandono de plantão.

Após a análise dessa categoria, foram selecionados alguns temas geradores das denúncias que exemplificam como essas ocorrências não éticas acontecem no exercício profissional da enfermagem (Quadro 1).

**Quadro 1:** Motivos geradores das denúncias - Processos éticos Coren-Santa Catarina, Brasil, 2017-2021

MOTIVOS DAS DENÚNCIAS
- Assédio moral
- Insubordinação
- Abuso de poder
- Abandono de plantão
- Desrespeito à hierarquia
- Confronto de competências
- Difamação de colegas
- Preconceito racial com o colega
- Ausência do setor sem autorização
- Falha na comunicação interpessoal
- Divulgação de fotos de pacientes em redes sociais
- Agressão física e verbal ao colega
- Desvio de medicamentos e insumos

A análise dessas ocorrências não éticas permitiu construir a Figura 1, mostrando a relação entre os motivos geradores das denúncias e as competências socioemocionais. Buscou-se demonstrar que as falhas geradoras das denúncias podem ocorrer com mais frequência quando o profissional não considera ou não têm fortalecidas as competências socioemocionais.

**Figura 1:** Motivos das denúncias relacionados às competências socioemocionais fundamentadas em Felcher et al^(11)^

A relação entre as competências socioemocionais e o exercício da enfermagem foi construída com base na análise dos principais motivos de denúncia que levaram profissionais a responderem a processos éticos no seu Conselho de classe. A proposta é utilizar essas situações como oportunidade de aprendizado, orientando os profissionais sobre a importância dos valores associados às competências socioemocionais diante dos desafios cotidianos da profissão. Quando incorporadas à prática, essas atitudes auxiliam significativamente na melhoria da qualidade do trabalho, das relações interpessoais e da tomada de decisão, de modo que favorecem uma assistência mais ética, segura e responsável.

As competências socioemocionais qualificam o desempenho profissional e as relações interpessoais na vida cotidiana. Considerando essa relação com a enfermagem, foram propostos alguns elementos que podem ajudar os profissionais a desenvolverem ou fortalecerem tais competências (Figura 2).

**Figura 2:** Elementos propostos para o desenvolvimento das competências socioemocionais

## DISCUSSÃO

Os erros na prática de enfermagem decorrem de fatores que merecem reflexão, em especial as condições de trabalho a que os profissionais estão expostos: sobrecarga de tarefas, déficit de pessoal, alta rotatividade e insuficiências na organização e gestão da assistência. Diante desses desafios estruturais e organizacionais, é preciso considerar estratégias que previnam erros geradores de danos a pacientes e suas famílias, incluindo abordagens formativas valorizando a dimensão ética do cuidado^(14)^.

No exercício da enfermagem, para além do domínio técnico-científico adquirido na formação, é essencial conhecer as leis, códigos e normas regulamentadoras da profissão. Entretanto, ao se analisar o conceito de Educação 5.0, emergem outros elementos igualmente relevantes para a formação ético-profissional, como empatia, responsabilidade social, colaboração e inteligência emocional. Essas competências, embora muitas vezes invisíveis aos currículos tradicionais, impactam diretamente a qualidade do cuidado e a postura ética dos profissionais.

Quando se analisa a relação entre as competências socioemocionais propostas pela Educação 5.0 e os motivos que geraram as denúncias dos processos éticos, fica claro o impacto que essas habilidades exercem sobre o trabalho da enfermagem. Embora não sejam quantificáveis nem possam ser registradas em currículo, tais competências fazem toda a diferença na vida pessoal e profissional de qualquer pessoa, já que são uma mistura de competência, habilidade e atitude^(9)^. Esses saberes são o diferencial do profissional^(15)^.

Entre as competências socioemocionais negligenciadas pelos profissionais nos processos analisados, destaca-se a Ética, considerada um instrumento social orientador do comportamento humano para viver bem em sociedade^(16)^. Pode-se dizer que todos os temas geradores das denúncias evidenciaram falhas nessa competência, no entanto, como exemplificação, optou-se por ilustrar cada habilidade com um tema cuja relação se mostrava mais clara.

A Ética enquanto competência nasce diante da oportunidade de escolha individual das ações, gerando uma responsabilidade ímpar^(17)^. Por exemplo, quando se confirmam denúncias relacionadas à divulgação de fotos de pacientes em redes sociais ou a outros temas citados no estudo, os profissionais estão ferindo a Ética.

Dessa forma, a cada tomada de decisão, é necessário realizar uma reflexão fundamentada na razão e nos princípios éticos, pois, caso contrário, o indivíduo tende a cometer infrações. Urge desenvolver programas educacionais que abordem os tipos de erros e suas causas, discutindo os cenários do problema e propostas de melhorias; a equipe de enfermagem, muitas vezes responsabilizada pelos erros, teme julgamentos e reações, o que a faz subnotificar os casos e falhar no seguimento das situações que incorreram em erros^(18)^.

Outra competência comprometida foi a empatia. Englobando capacidades cognitivas e emocionais, ela é crucial na enfermagem, sobretudo para compreender e se conectar com os sentimentos dos outros^(19)^. Sua ausência pode levar a ações prejudiciais, como o abandono de plantão ou a divulgação indevida de fotos de pacientes nas redes sociais. Outros autores ressaltam a empatia como vital para a saúde, enfatizando a necessidade de reformas na cultura organizacional das instituições de saúde para melhorar a performance e o bem-estar no ambiente de trabalho^(20)^.

Houve falha também de habilidade na comunicação e resolutividade de problemas. Esses problemas podem comprometer a segurança dos pacientes e a harmonia da equipe. Associamos o comprometimento dessa competência com o motivo “Falha na comunicação interpessoal”. Por exemplo, a passagem de plantão é um momento decisivo para a comunicação entre equipes de enfermagem, pois a troca de informações efetiva garante a continuidade e a qualidade da assistência, fundamentais para a segurança do paciente^(21)^.

Assim, a comunicação eficaz não só é essencial para promover a segurança do paciente, mas também para resolver conflitos e proporcionar flexibilidade e trabalho em equipe^(22)^. A experiência e os valores profissionais, aliados às habilidades de desenvolver estratégias focadas na salvaguarda do paciente, formam o cerne da tomada de decisões no cuidado de enfermagem^(23)^.

O trabalho em equipe é outra competência socioemocional que condiciona o bom desempenho dentro desse novo modelo de educar. No presente estudo, tal habilidade foi comprometida quando o profissional abandonou o plantão. Alguns autores^(22,24)^ destacaram o relacionamento interpessoal e o trabalho em equipe na área de enfermagem como fatores fundamentais para o trabalho; quando articulados com estratégias de gestão eficazes, não só enfrentam desafios e melhoram resultados, mas também aumentam a motivação e o desempenho do pessoal. Eles mostram que o trabalho em equipe subjaz à manutenção do cuidado seguro, promovendo a coesão bem como a partilha de conhecimentos e objetivos, o que diretamente melhora a qualidade do atendimento e a satisfação tanto da equipe quanto dos pacientes.

Problemas na habilidade de controle e gerenciamento das emoções também foram associados aos motivos geradores das denúncias, como nos casos de abuso de poder. A gestão eficaz das emoções é destacada como uma competência-chave na enfermagem, especialmente em contextos de abuso de poder. A inteligência emocional - entendida como a habilidade de compreender e gerenciar sentimentos próprios e alheios - tem papel decisivo na resolução de conflitos, alcance de objetivos e manutenção de relações interpessoais saudáveis no ambiente de trabalho^(25,26)^. Na liderança em enfermagem, torna-se imprescindível aplicar essa capacidade para mitigar o desgaste emocional da equipe, promovendo uma prática de cuidado compassiva e efetiva^(27)^.

É necessário investir em ações de educação permanente que capacitem os enfermeiros para gerenciar conflitos. Salienta-se a importância da capacitação para o desenvolvimento de competências como relacionamento interpessoal e comunicação, além da discussão dessa temática ainda na graduação^(28)^.

A diversidade, reconhecida como uma habilidade socioemocional essencial, destaca-se na enfermagem especialmente em casos de denúncias de preconceito racial. Refletindo o conceito de um espectro amplo de diferenças humanas, abrange aspectos profissionais, geracionais, capacidades, orientações sexuais e crenças religiosas^(29)^. Impulsionada pela busca de uma sociedade mais justa, a diversidade vem sendo cada vez mais reconhecida em diversos contextos, promovendo mudanças significativas^(30)^.

Discutir diversidade implica explorar as múltiplas facetas da experiência humana, incluindo características pessoais, culturais e históricas. Essa reflexão nos ajuda a compreender melhor a nós mesmos e aos outros, conferindo o devido respeito às diferenças individuais. Profissionais que valorizam a diversidade participam da construção de um ambiente mais inclusivo e ético em sua prática^(30)^.

No século XXI, os programas de educação devem formar profissionais reflexivos, críticos e capazes de provocar mudanças, não apenas ocupando um espaço, mas também sendo verdadeiros agentes de transformação^(11)^. Tanto quanto ao avanço tecnológico, o futuro da sociedade está intrinsecamente ligado ao desenvolvimento de práticas éticas robustas^(31)^.

Nesse sentido, a Educação 5.0 mostra-se como um modelo inovador na formação de pessoas para entrada no mercado de trabalho, com aquisição das competências socioemocionais necessárias para serem profissionais colaborativos e atuantes em prol do coletivo, formando uma sociedade mais altruísta^(32)^. Embora algumas já sejam conhecidas nas esferas profissional e pessoal, essas habilidades passam a assumir um lugar de destaque nesse novo cenário.

Sob essa perspectiva, é prudente capacitar os profissionais para uma prática ética, criativa, reflexiva e resolutiva, considerando que tais competências desenvolvem conhecimento, habilidade e atitude, fazendo a diferença na conduta dos profissionais. O conhecimento sozinho não é suficiente: precisa estar presente na habilidade e na atitude dos profissionais.

Com esse olhar, foram propostos alguns elementos para fortalecer as competências socioemocionais: valorização de habilidades individuais, discussão em equipe, liderança compartilhada, cultura organizacional não punitiva, compartilhamento de saberes e educação permanente.

Quando as habilidades individuais são valorizadas, os profissionais sentem-se importantes dentro da equipe, de forma que se estimula a troca de saberes e o trabalho em equipe. Esse elemento associa-se à discussão entre as equipes. Ela desempenha um papel fundamental nos serviços de saúde, ao promover a união dos profissionais e ampliar a visão dos problemas, pois cada profissional possui uma opinião diferente, contribuindo para a melhoria dos serviços.

Já no que tange à liderança, é preciso que o líder escuta sua equipe, compartilhe ideias e leve em consideração as opiniões do grupo. Assim, ele certamente tomará decisões mais assertivas e terá o respeito e admiração da sua equipe, promovendo um ambiente de trabalho saudável e empático. Em um estudo realizado para avaliar a segurança dos pacientes em um hospital filantrópico de Minas Gerais, observou-se que a comunicação entre os profissionais e a capacidade dos líderes de ouvir a sua equipe favorecem uma assistência mais segura^(33)^.

Outro elemento proposto é a cultura organizacional não punitiva nos serviços de saúde. Ela cria um ambiente propício para que os eventos adversos sejam registrados, e o conhecimento desses erros oferece a possibilidade de implementar melhorias, tais como a criação de protocolos e mudança na rotina dos serviços, por exemplo. Em um estudo realizado para avaliar a cultura de segurança dos pacientes na percepção dos profissionais de enfermagem, destacou-se a importância de os erros não serem tratados de uma forma punitiva pelos gestores, pois o clima de punição resulta no medo de notificar o erro. Em vez disso, os autores recomendam que os erros sejam analisados de forma educativa^(34)^.

A educação permanente também é um elemento proposto para fortalecer as competências. É vista como uma das principais estratégias de redução de erros na enfermagem. A educação é o elemento que fortalece todos os outros, pois o conhecimento é poder: é poder discutir melhor em equipe, é poder liderar melhor, compartilhar e multiplicar os saberes, é saber valorizar as habilidades individuais.

### Limitações do estudo

Os documentos acessados não disponibilizavam informações detalhadas sobre os locais específicos de ocorrência dos fatos relatados nos processos éticos - se eram hospitais, unidades básicas de saúde, clínicas ou outros serviços. Esse dado poderia contribuir para análises mais aprofundadas sobre contextos institucionais e suas possíveis influências nas condutas éticas. Além disso, o caráter documental da pesquisa restringe a compreensão da intencionalidade ou das circunstâncias subjetivas envolvidas nos episódios analisados.

### Contribuições para a área da enfermagem, saúde ou política pública

Os achados deste estudo contribuem para fortalecer a educação ético-profissional na enfermagem ao identificarem, com base em processos éticos reais, elementos formativos alinhados às competências socioemocionais propostas pela Educação 5.0. Esses resultados oferecem subsídios tanto para a qualificação da formação acadêmica quanto para a implementação de estratégias de educação permanente nos serviços de saúde. Além disso, podem orientar políticas institucionais e ações de gestão voltadas à promoção de uma prática profissional mais ética, segura e humanizada.

## CONSIDERAÇÕES FINAIS

Os motivos geradores dos processos éticos analisados neste estudo nos remetem à necessidade de refletir sobre quais conhecimentos, habilidades e atitudes ainda se encontram fragilizados ou carecem de aperfeiçoamento. Essas reflexões estimulam o desenvolvimento de medidas para reduzir condutas não éticas por parte dos profissionais de enfermagem. Os achados também reforçam a importância de se investir, de forma contínua e estruturada, na educação ético-profissional ao longo da formação e do exercício profissional.

É inegável o impacto que a formação profissional, a educação permanente e o domínio sobre as leis, códigos, normas e resoluções exercem na prática profissional, influenciando diretamente a forma de comunicar, de executar procedimentos e de tomar decisões clínicas e éticas. Nesse sentido, as competências socioemocionais propostas pela Educação 5.0, discutidas neste trabalho, introduzem habilidades essenciais à formação, contribuindo para uma atuação mais ética, colaborativa e sensível às demandas contemporâneas da profissão.

Assim, o fortalecimento de competências como ética, empatia, trabalho em equipe, controle e gerenciamento das emoções, comunicação, resolutividade de problemas e valorização da diversidade pode favorecer a construção de práticas mais seguras, reflexivas e responsáveis, fundamentadas na Ética do Cuidado. Profissionais que desenvolvem essas competências tendem a exercer o cuidado com maior consciência de seu papel social, promovendo relações interpessoais saudáveis e contribuindo ativamente para a qualificação da enfermagem e dos serviços de saúde.

## Data Availability

Os dados de pesquisa só estão disponíveis mediante solicitação.
